# Centralized Digital Surveillance for Abdominal Aortic Aneurysm Detection, Longitudinal Tracking, and Management Within an Integrated Health System: Retrospective Cohort Study

**DOI:** 10.2196/93443

**Published:** 2026-08-19

**Authors:** Evan J Ryer, Gregory G Salzler, Matthew Goldfarb, Anthony J Lewis, Yatin B Mehta, Ian R Dinsmore, Tooraj Mirshahi, Elliot G Mitchell, Grant DeLong, James R Elmore

**Affiliations:** 1 Department of Vascular and Endovascular Surgery, Geisinger Medical Center, 100 N. Academy Ave, Danville, PA, 17822, United States, 1 570 271 6369; 2 Division of Critical Care Medicine, Geisinger Medical Center, Danville, PA, United States; 3 Department of Genomic Health, Geisinger Health System, Danville, PA, United States; 4 Geisinger AI, Danville, PA, United States

**Keywords:** abdominal aortic aneurysm, AI, natural language processing, surveillance, population health, imaging follow-up

## Abstract

**Background:**

Incidental detection of abdominal aortic aneurysms (AAAs) has increased with widespread cross-sectional imaging, while traditional surveillance remains fragmented and clinician-dependent. Real-world descriptions of centralized digital surveillance programs combining structured electronic health record (EHR) queries with natural language processing (NLP) of radiology reports remain limited.

**Objective:**

This study aimed to describe the design, implementation, and operational performance of the System to Track Abnormalities of Importance Reliably (STAIR), a centralized digital surveillance program combining structured EHR queries, an internally developed NLP model, clinician referrals, and automated lost-to-follow-up detection to identify, track, and manage patients with AAAs across a large integrated health system.

**Methods:**

This retrospective cohort study included all patients enrolled in the STAIR AAA surveillance program from December 2022 through December 2024. Patients were identified via 4 pathways: an internally developed NLP model applied to radiology reports, EHR problem-list queries, clinician referrals, and automated lost-to-follow-up queries. All cases underwent standardized centralized clinical review, and surveillance intervals were assigned using guideline-informed institutional protocols. Administrative status and ongoing surveillance were assessed through April 2026. Outcomes were descriptive. Cohort integrity was evaluated against an independent, automated, computed tomography (CT)–based measurement system among patients with CT imaging.

**Results:**

A total of 8464 patients were enrolled (mean age 77.1, SD 8.8 y; male: n=6516, 77.0%; and White: n=83119, 8.2%). Identification was predominantly automated via problem-list queries (n=4994, 59.0%) and radiology NLP (n=2454, 29.0%), with clinician referral (n=592, 7.0%) and lost-to-follow-up queries (n=423, 5.0%). Following centralized review, most patients were assigned guideline-based duplex surveillance (biennial: 45.3%; 5-year: 9.5%); 20.6% were referred for vascular surgery evaluation, and another 20.6% had prior AAA repair at enrollment. Among all 8464 enrolled patients, 4188 (49.5%) remained under active surveillance as of April 2026, and 2709 (32.0%) had transitioned to management outside the health system. Duplex ultrasonography was the predominant surveillance imaging modality (6122/6951 patients, 88.1%); 351 elective AAA repairs were performed system-wide during the study period, including 260 in the enrolled cohort.

**Conclusions:**

In a large integrated health system, this centralized digital surveillance infrastructure was operationally feasible and supported large-scale, structured identification, guideline-based surveillance and management assignment, and documented administrative status through April 2026 for all 8464 enrolled patients, including those who could not be reached despite repeated outreach. These descriptive findings establish operational feasibility only and characterize a scalable, workflow-focused approach to population-level AAA surveillance and management that emphasizes structured clinical oversight rather than autonomous AI decision-making; clinical effectiveness is not demonstrated by this design.

## Introduction

### Background

Abdominal aortic aneurysm (AAA) surveillance has changed substantially in the past 2 decades. Early population-screening trials reported aneurysm prevalence exceeding 4%, supporting one-time ultrasound screening in selected populations [1,2]. Contemporary cohorts have demonstrated a markedly lower prevalence, often between 0.5% and 1.5%, reducing the yield and cost-effectiveness of traditional screening strategies [3,4]. Concurrently, the widespread use of cross-sectional imaging has led to frequent incidental detection of AAAs in clinical contexts not directly connected to vascular care [5].

Incidental AAAs are underreported or not acted upon in up to half of cases, and many patients never undergo structured surveillance after an initial radiographic finding [6]. These system-level failures contribute to delayed recognition of aneurysm progression, late referral, emergency presentation, and increased risk of rupture [1,5]. Most health systems lack reliable mechanisms to identify AAAs across care environments and to ensure imaging at guideline-concordant intervals. Surveillance often depends on individual clinicians, variable documentation practices, and fragmented follow-up, with transitions between inpatient, outpatient, and external providers further increasing loss to follow-up [7-9].

### Prior Work

Centralized digital surveillance models have been described for other incidental radiologic findings [10]. Advances in AI, particularly in natural language processing (NLP) of radiology reports and automated tracking systems, provide an opportunity to address these gaps at scale. Prior studies have demonstrated the feasibility of AI-based aneurysm detection and measurement using radiological texts and imaging data [11,12]. More recently, several groups have reported single-institution NLP-based AAA surveillance efforts, including nurse-navigator-driven programs that pair commercial NLP with electronic health record (EHR) review to identify patients not receiving appropriate surveillance [13], programs reporting surveillance-adherence rates ranging from approximately 39% to 68% [14,15], and transformer-based architectures applied to flagging and measuring aortic diameters in radiology reports [16]. Large language models have shown high accuracy for extracting aortic information directly from imaging reports without task-specific training [17]. In contrast to these prior single-detector or single-pathway efforts, real-world descriptions of population-scale AAA surveillance programs that integrate multiple parallel identification pathways with centralized clinical review and documented administrative status tracking, particularly those operating across an entire integrated health system rather than at a single academic site, remain limited.

### Study Aim

This study aimed to describe the design, implementation, and operational performance of the System to Track Abnormalities of Importance Reliably (STAIR), a centralized digital surveillance program that combines structured EHR queries with an internally developed NLP model applied to radiology reports and was implemented within a large, integrated health system to identify AAAs across multiple pathways, assign guideline-based surveillance and management, and ensure longitudinal administrative status tracking. We hypothesized that pairing automated detection with a structured, centralized clinical review would support large-scale case identification and documented administrative status tracking at a population scale.

## Methods

### Study Design and Setting

**Figure 1.**
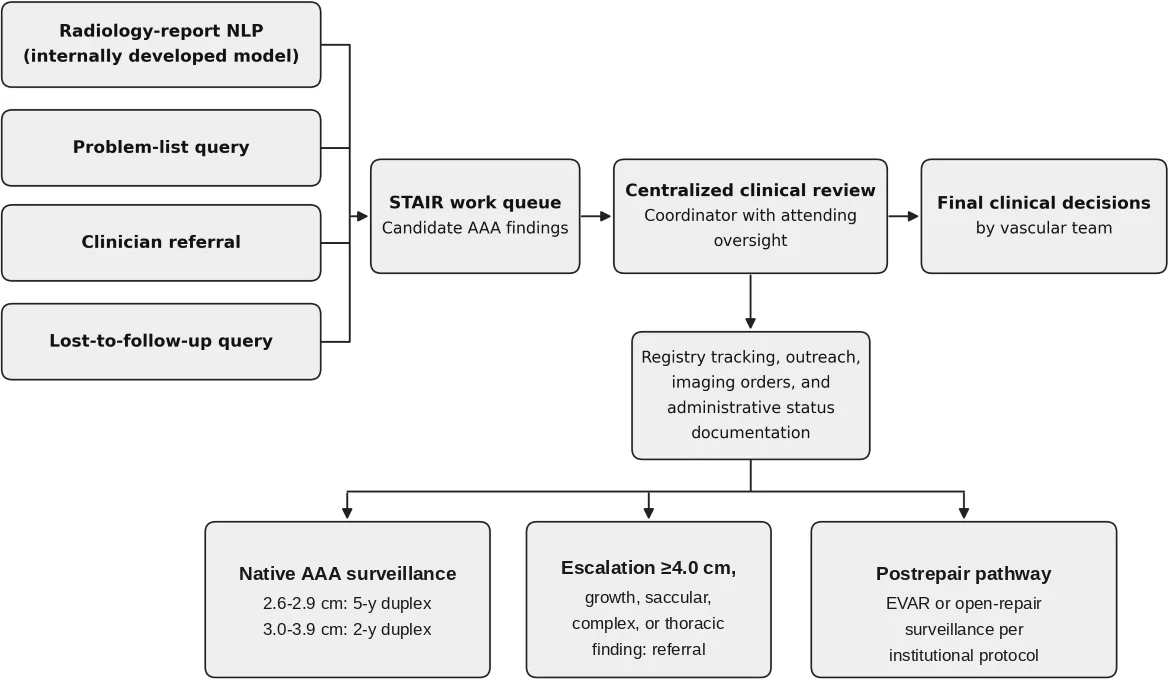
STAIR AAA surveillance workflow, Geisinger Medical Center, from December 2022 onward. AAA: abdominal aortic aneurysm; EVAR: endovascular aneurysm repair; NLP: natural language processing; STAIR: System to Track Abnormalities of Importance Reliably.

This is a retrospective cohort study of all patients enrolled in the STAIR AAA surveillance program within a large integrated health system from program implementation in December 2022 through December 2024. The program is operated by the Department of Vascular and Endovascular Surgery and serves a predominantly rural population across central and northeastern Pennsylvania. Enrollment in the STAIR registry reflects prospective operational entry as part of routine clinical care following program initiation in December 2022, with the retrospective inclusion of patients identified through automated detection at the time of implementation; this study is a retrospective secondary analysis of this operational quality improvement registry (see Ethical Considerations). A sample workflow of the STAIR AAA program is illustrated in Figure 1. Preliminary operational data from the program were presented at the Eastern Vascular Society 2025 Annual Meeting [18].

Candidate AAA findings enter the STAIR work queue through 4 parallel identification pathways: automated radiology-report screening using an internally developed NLP model, structured EHR problem-list query, direct clinician referral, and automated lost-to-follow-up query. The work queue is triaged by the vascular surgery coordinator under physician oversight (centralized clinical review), and final clinical decisions are made by the vascular team. Each enrolled patient then enters registry tracking with outreach, imaging orders, and administrative status documentation, and is assigned to 1 of 3 downstream pathways: native AAA surveillance (2.6‐2.9 cm → duplex ultrasonography every 5 years; 3.0‐3.9 cm → duplex ultrasonography every 2 years); escalation (≥4.0 cm, interval growth, saccular or complex morphology, or thoracic finding → vascular surgery referral); or postrepair pathway (post–endovascular aneurysm repair [EVAR] or post–open-repair surveillance per institutional protocol). Thoracic and complex aortic findings encountered during NLP screening are routed for vascular surgery referral and are not enrolled in native AAA interval surveillance. At the age of 90 years, patients are transitioned from active STAIR surveillance to their primary care provider for continued surveillance as appropriate.

### Ethical Considerations

This study was reviewed by the Geisinger Institutional Review Board (protocol: 2025‐0177; reference: 060066) and was determined to be exempt on March 5, 2025, because of minimal risk and the use of deidentified data. The STAIR program was implemented in December 2022 as a clinical and operational quality-improvement initiative within routine vascular surgery care; the analyses reported here constitute retrospective secondary analyses of the registry data generated by that program, and the March 2025 institutional review board exempt determination covers these retrospective analyses. No prospective human participant research was conducted prior to ethical review. Given the exempt determination, additional informed consent was not required; original consent for the use of electronic health record data for research is covered by Geisinger’s general consent for treatment and research data use. All study data were deidentified before analysis, and no individually identifiable information appears in the paper. No participant compensation was provided because the study consisted of secondary analysis of clinical data. The internally developed NLP model operated entirely within Geisinger’s secured information technology environment under standard institutional data-governance arrangements; no protected health information was transmitted to an external vendor for case identification during the study period.

### Program Framework

The STAIR program was designed to centralize AAA surveillance by integrating automated case identification, registry-based tracking, and guideline-based clinical review. AI-derived outputs were used exclusively to support case identification and workflow prioritization and did not independently direct clinical decision-making. All surveillance assignments, referrals, and management decisions were made through standardized central clinical review by a vascular surgery coordinator under the supervision of a vascular surgeon. Discrepancies between AI-identified information and clinical assessment were resolved by the vascular surgery team. The program excluded thoracic aortic aneurysms from longitudinal surveillance; thoracic or saccular aneurysms identified during NLP screening were routed to immediate vascular surgery referral and were not enrolled in the AAA Surveillance Registry. Patients who reached 90 years of age during follow-up were transitioned to clinician-directed management without further routine STAIR surveillance, consistent with institutional practice for advanced age and prohibitive comorbidity. The age-90 transition reflects a deliberate de-escalation of protocolized, population-level surveillance rather than a transfer of responsibility for managing actionable disease: repair after age 90 is offered only to selected patients after extensive shared decision-making with the patient and family, and those with concerning findings may be rereferred for vascular surgery evaluation at any time. The transition is, therefore, distinct from, and not in tension with, the program’s aim of reliably ascertaining actionable aneurysms across the broader at-risk population in whom such findings might otherwise be missed.

### Patient Identification

Patients entered the STAIR registry through 4 predefined pathways. First, an internally developed NLP model (described in the following section) continuously analyzed institutional radiology reports for aneurysm-related terminology and diameter measurements. Second, structured EHR problem-list queries identified patients with documented AAA diagnoses. Third, clinicians submitted direct referrals for newly identified or previously known aneurysms. Fourth, automated lost-to-follow-up queries identified patients who exceeded recommended surveillance intervals based on guideline-defined thresholds.

### Case-Identification Model (NLP)

AAA case identification used an NLP model developed internally at Geisinger with iterative clinical input. The model was built in Python (Python Software Foundation) using SentenCy, an open-source spaCy-based library developed by our group to enable sentence-level named-entity recognition, in conjunction with the scispaCy biomedical NLP library [19]. Custom keyword and regular-expression rules were tuned for aneurysm-related terminology and negation detection and applied to institutional radiology reports to flag candidate AAA findings, while reports describing a normal abdominal aortic diameter were excluded. The model identified candidate findings and populated program-specific work queues; it did not make autonomous clinical diagnoses or treatment recommendations. This internally developed model was used for case identification throughout the study period.

The model was validated against a Geisinger-specific reference standard. A training dataset was assembled from STAIR program cases (N=4076), from which a curated set of 300 radiology reports was labeled; through 3 rounds of iterative review with the clinical team, the model was refined and gold-standard labels were established. In a held-out gold-standard dataset of 99 imaging studies, the model achieved a precision (positive predictive value) of 0.98, a recall (sensitivity) of 0.90, and a specificity of 0.96. The confusion matrix comprised 64 true positives, 1 false positive, 27 true negatives, and 7 false negatives (71 positive and 28 negative studies); Wilson-score 95% CIs were 0.92 to 1.00 for precision, 0.81 to 0.95 for recall, and 0.82 to 0.99 for specificity. Two features of the evaluation set limit direct generalization to a consecutive screening population. First, negative cases were underrepresented, so the prevalence of AAA findings in the set (71/99) was substantially higher than in unselected imaging, and because sensitivity and specificity are intrinsic test properties independent of disease prevalence, whereas positive predictive value increases with prevalence, the precision estimate is likely optimistic (an upper bound) relative to a lower-prevalence screening population. Second, the gold-standard studies were not a consecutive sample but were drawn from reports surfaced or adjudicated within the STAIR workflow, which enriched the set for borderline, difficult-to-classify cases and underrepresented the many imaging studies containing no AAA-related language at all; consequently, all 3 metrics may not transfer directly to the full imaging population, and the direction of any bias in sensitivity and specificity cannot be assumed. External validation on a consecutively sampled imaging cohort is therefore needed. The model was developed and retuned iteratively over the course of the program, with detection performance improving across successive iterations; the validation reported here reflects the most recent iteration. The model was used to identify candidate findings and populate program-specific work queues aligned with the STAIR workflow; final determination of registry inclusion, surveillance-interval assignment, and referral remained the responsibility of the centralized vascular surgery review team under attending supervision.

### Centralized Clinical Review and Surveillance Assignment

Each identified case underwent standardized review by a trained vascular surgery coordinator, who evaluated prior imaging, aneurysm size, and relevant clinical context. Surveillance thresholds were 2.6 to 2.9 cm (5-y duplex), 3.0 to 3.9 cm (2-y duplex), and ≥4.0 cm (vascular surgery referral). The program adopted surveillance intervals more conservative than those recommended in the Society for Vascular Surgery (SVS) and the European Society for Vascular Surgery (ESVS) guidelines [20,21]: biennial duplex was used for 3.0 to 3.9 cm aneurysms rather than triennial surveillance, and all patients with maximal aortic diameter ≥4.0 cm were referred directly for vascular surgery evaluation rather than continuing serial imaging at 12- or 6-month intervals. This approach was selected to standardize centralized review pathways and ensure timely surgical assessment as aneurysms approached operative thresholds. Patients meeting guideline-based criteria for repair or demonstrating concerning interval growth were referred for vascular surgery evaluation. Patients with incomplete or outdated imaging were scheduled for expedited duplex ultrasonography. All surveillance assignments followed standardized protocols and were made under the supervision of a vascular surgeon to ensure consistency.

### Surveillance of Patients With Prior Aneurysm Repair

Patients entering the registry with a history of EVAR or open AAA repair were assigned postoperative surveillance schedules consistent with the SVS and ESVS guidelines [20,21]; these surveillance schedules remain consistent with the 2024 ESVS update [22]:

- Post-EVAR surveillance: Initial imaging consisted of computed tomography angiography (CTA) at 1 month following repair or duplex ultrasonography combined with noncontrast computed tomography (CT) in patients for whom contrast was contraindicated. Repeat imaging was performed at 12 months using either CTA or duplex when the 1-month study showed a stable sac and no endoleak. After the first year, patients with a stable sac and no endoleak were transitioned to annual duplex surveillance. Patients with a type II endoleak and a stable sac underwent duplex every 6 to 12 months. Patients with sac growth or unresolved endoleak underwent CTA and were treated as clinically indicated, with intervention thresholds determined by the vascular surgeon [21].
- Post–open AAA repair surveillance: Patients with a prior open AAA repair were scheduled for a baseline CT of the chest, abdomen, and pelvis at 5 years post repair. Subsequent imaging was performed only if the patient was symptomatic or if there was clinical concern for a para-anastomotic aneurysm [20].
- Postoperative patients followed the same registry-tracking workflow as patients under primary surveillance: Centralized clinical review confirmed adherence to scheduled imaging, identified patients requiring escalation, and recorded the administrative status when postoperative surveillance transitioned to out-of-system management or another status.

### Longitudinal Tracking and Administrative Status Determination

Registry tracking incorporated automated reminders and coordinator outreach to support completion of scheduled imaging. Patients were classified as unreachable only after multiple documented contact attempts using available communication modalities over a predefined interval, consistent with institutional follow-up standards. Tracking continued until a predefined administrative status or clinical outcome was recorded, including continued surveillance, referral for endovascular or open repair, transfer of care outside the health system, patient refusal, death, or inability to establish contact despite repeated attempts. For patients transitioning to management outside the health system, a structured handoff letter was generated and sent to the receiving primary care provider, documenting the patient’s current STAIR surveillance assignment and recommended follow-up imaging. Patients reaching a defined administrative status were documented accordingly. Patients classified as remaining under active surveillance were those who remained enrolled in the STAIR program at the April 2026 data cutoff and had not reached any terminal administrative status (transfer of care outside the health system, release from protocolized surveillance, death, refusal of further follow-up, or inability to establish contact). This category was, therefore, defined as the complement of the terminal status categories rather than by a separate positive registry flag and comprises patients continuing under scheduled guideline-based surveillance imaging and coordinator outreach.

### Cohort Definitions

Patients within the system undergoing AAA repair during the study period were classified as “cohort” if they were enrolled in the STAIR Surveillance Registry at the time of repair (ie, the patient had been previously identified through 1 of the 4 enrollment pathways and had completed at least one centralized review before the operative encounter). Patients undergoing repair within the health system who had not been previously enrolled in STAIR at the time of presentation, typically those presenting acutely or transferred from external facilities, were classified as “noncohort.” This classification was determined administratively at the time of operative encounter and was based on registry status alone, independent of postoperative enrollment.

### Outcome Measures and Validation

Outcomes were descriptive and included sources of patient identification, surveillance assignment frequencies, administrative status categories, surveillance imaging utilization, and elective aneurysm repair activity. For validation purposes, STAIR cohort identification was compared with the results from an independent automated CT-based abdominal aortic diameter measurement system operating outside the STAIR detection workflow. Validation analyses were limited to the 2894 patients with available CT imaging and were intended to confirm cohort integrity rather than to estimate diagnostic accuracy or population prevalence. Cohort integrity for patients evaluated solely by ultrasonography could not be independently verified by the CT-based system; for these patients, registry inclusion relied on standardized, centralized clinical review of the original ultrasound reports and source documentation.

### Statistical Analysis

Analyses were descriptive. Continuous variables were summarized as mean (SD) and median (IQR); categorical variables were summarized as counts and percentages. Denominators reflected the total number of patients with nonmissing data for the variable in question. Follow-up duration was summarized as median (IQR) from the date of registry enrollment until the date of the last observable postenrollment EHR contact and was reported for the subset of patients with any postenrollment EHR activity. Data cutoff for postenrollment follow-up analyses was April 2026; administrative status and EHR-contact follow-up were updated through this date. Surveillance imaging-modality usage was analyzed separately using a structured registry extract generated in December 2025; these represented 2 distinct data pulls. Operative volumes (elective and ruptured AAA repairs) were ascertained over the enrollment period (extending from the fourth quarter of 2022 through the fourth quarter of 2024) to maintain a consistent observation window. Engagement intensity was characterized by comparing the duration of observable EHR history, the absolute number of distinct EHR events, and the time-normalized EHR event rate before versus after STAIR enrollment, restricted to the subset of patients with observable EHR activity in both the pre- and postenrollment periods. Surveillance imaging-modality usage was derived from a structured registry data extract (extract dated Dec 2025) comprising 8405 enrolled patients with available structured demographic and imaging records; modality proportions were reported among the 6951 of these patients with a documented surveillance imaging study, with patients lacking a recorded surveillance study (eg, those referred directly to vascular surgery, undergoing repair, or reaching an early administrative status) excluded from the denominator. The 59 enrolled patients not represented in this extract (8464 vs 8405) lacked complete structured demographic records as of the extract date and were therefore not returned by the demographics query.

## Results

### Baseline Characteristics and Patient Identification

A total of 8464 patients were included in the STAIR AAA Surveillance Registry (Table 1). The mean age was 77.1 (SD 8.8) years, and the median age was 77.7 (IQR 70.9‐84.0) years. Among the 8464 enrolled patients, 6516 (77.0%) were male and 1948 (23.0%) were female. The cohort was predominantly White (8311/8464, 98.2%) and non-Hispanic (8370/8464, 98.9%). Medicare was the most common primary insurance, including traditional Medicare (5552/8464, 65.6%) and Medicare Advantage (1434/8464, 16.9%). Most patients were former (4920/8464, 58.1%) or current (2307/8464, 27.3%) smokers.

Patients were most commonly identified through automated processes, including problem-list interrogation (4994/8464, 59.0%) and radiology NLP (2454/8464, 29.0%). Additional patients were identified through direct clinician referral (592/8464, 7.0%]) or automated lost-to-follow-up queries (423/8464, 5.0%) (Table 1).

Median postenrollment follow-up duration was 20.2 (IQR 9.5‐28.9) months, reported for the 7018 patients (82.9% of the enrolled cohort) with observable postenrollment EHR activity. The remaining 1446 patients had no postenrollment EHR contact at the time of the registry query, typically reflecting early administrative statuses such as death, transfer of care outside the health system, or an unreachable status before any postenrollment touchpoint.

**Table 1.** Baseline characteristics, identification sources, and in-study AAA^a^ repair activity of 8464 patients enrolled in the STAIR^b^ AAA Surveillance Registry, Geisinger Health System, December 2022 through December 2024^c^.

Characteristics	Values
Age (y), mean (SD)	77.1 (8.8)
Age (y), median (IQR)	77.7 (70.9‐84.0)
Sex, n (%)	
Male	6516 (77.0)
Female	1948 (23.0)
Race, n (%)	
White	8311 (98.2)
Black or African American	118 (1.4)
Asian	16 (0.2)
American Indian or Alaska Native	10 (0.1)
Native Hawaiian or Other Pacific Islander	5 (0.1)
Other	4 (<0.1)
Ethnicity, n (%)	
Not Hispanic or Latino	8370 (98.9)
Hispanic or Latino	94 (1.1)
Insurance type, n (%)	
Medicare (traditional)	5552 (65.6)
Medicare Advantage	1434 (16.9)
Commercial insurance^d^	745 (8.8)
Self-pay	232 (2.7)
Military or Veterans Affairs	117 (1.4)
Medicaid replacement	99 (1.2)
Other	226 (2.7)
Unknown or not recorded	59 (0.7)
Smoking status, n (%)	
Former	4920 (58.1)
Current	2307 (27.3)
Never	1089 (12.9)
Unknown or not assessed	148 (1.7)
AAA repair performed during study period among enrolled STAIR cohort patients, n (%)	
Endovascular aneurysm repair	212 (2.5)
Open aneurysm repair	48 (0.6)
Patient identification source, n (%)	
Problem list	4994 (59.0)
Radiology NLP^e^ detection	2454 (29.0)
Direct clinician referral	592 (7.0)
Lost-to-follow-up query	423 (5.0)
Other or unclassified	1 (<0.1)

a AAA: abdominal aortic aneurysm.

b STAIR: System to Track Abnormalities of Importance Reliably.

c Baseline demographic and clinical characteristics, identification sources, and in-study AAA repair activity of 8464 patients enrolled in the STAIR Abdominal Aortic Aneurysm Surveillance Registry from the program’s implementation in Dec 2022 through Dec 2024 at Geisinger, an integrated rural health system in central and northeastern Pennsylvania. Where applicable, subgroups include explicit “Unknown or not recorded” rows so each category counts sums to 8464. Percentages are rounded to 1 decimal place.

d Includes employer-based and Blue Cross Blue Shield plans.

e NLP: natural language processing.

### Confirmation of Cohort Integrity

Cohort integrity was evaluated using the independent CT-based diameter measurement system. Among 2894 patients with available CT-derived measurements, 2801 (96.8%) had a maximal abdominal aortic diameter ≥2.6 cm and 2341 (80.9%) met the aneurysm threshold of ≥3.0 cm. The remaining 93 patients (3.2%) had mildly ectatic aortas with maximal diameter of less than 2.6 cm on CT and were not explicitly described as aneurysmal in the original radiology reports; these patients were retained in the registry pending repeat surveillance imaging. Within the triage pathways in Table 2, these 93 patients are included among the 4637 patients assigned to duplex ultrasonography surveillance and do not constitute an additional triage category; the surveillance interval was assigned after centralized review of the original report, prior measurements, and clinical context. The 8 patients with aortic diameter of less than 2.6 cm shown in Table 2 represent a separate subset whose surveillance-modality measurement (predominantly duplex) confirmed subthreshold diameter and who were therefore released from active surveillance.

**Table 2.** Initial surveillance or management assignment for 8464 enrolled patients (STAIR AAA^a^ Registry, December 2022 through December 2024)^b^.

Pathway assigned	Patients, n (%)
Duplex ultrasonography every 2 years	3833 (45.3)
Referral to vascular surgery	1744 (20.6)
History of prior EVAR^c^ at enrollment	1337 (15.8)
Duplex ultrasonography every 5 years	804 (9.5)
History of prior open-repair at enrollment	407 (4.8)
Immediate duplex required	313 (3.7)
Aortic diameter <2.6 cm (below surveillance threshold)	8 (0.1)
Other or administrative classification	18 (0.2)
Total	8464 (100)

a STAIR AAA: System to Track Abnormalities of Importance Reliably Abdominal Aortic Aneurysm.

b Guideline-based initial surveillance or management assignments following standardized, centralized clinical review for 8464 patients enrolled in the STAIR AAA Registry from Dec 2022 through Dec 2024. Categories include an explicit “Other or administrative classification” row so that total counts sum to 8464. The 1744 prior-repair patients (1337 prior EVAR and 407 prior open-repair) are shown as postrepair surveillance pathway categories and are distinct from the 1744 patients referred for new vascular surgery evaluation; the numerical equality between these 2 categories is coincidental.

c EVAR: endovascular aneurysm repair.

### Surveillance and Management Assignment

Following centralized clinical review, 1744/8464 (20.6%) patients were referred for vascular surgery evaluation (Table 2). Surveillance imaging was assigned to 4637 patients, including biennial duplex for 3833 (82.7% of those assigned surveillance) and 5-year duplex for 804 (17.3%). Immediate imaging was required for 313 patients (3.7%) because prior studies were outdated or inadequate. Prior aneurysm repair was identified at enrollment among a separate cohort of 1744 patients, including 1337 (15.8%) with prior EVAR and 407 (4.8%) with prior open repair; these postoperative patients were tracked under the postrepair surveillance pathway rather than as new vascular surgery referrals, and the numerical equality with the 1744 new vascular surgery referrals is coincidental. Eight patients (0.1%) had aortic diameters below surveillance thresholds and required no further follow-up; an additional 18 patients had administrative classifications captured under “other” and are presented in the “Other or administrative classification” pathway in Table 2 to ensure subgroup counts sum to 8464.

### Administrative Status Through April 2026

Each of the 8464 enrolled patients was assigned a single documented administrative status as of April 2026 (Table 3). A total of 4188 patients (49.5%) remained under active surveillance; 2709 (32.0%) had transitioned to management outside the health system, most commonly through primary care follow-up; 623 (7.4%) required no further protocolized STAIR surveillance because of advanced age (including transition from protocolized surveillance at the age of 90 y) or prohibitive comorbidities; 387 (4.6%) died; 293 (3.5%) declined further participation; and 264 (3.1%) were classified as “unable to reach” despite repeated documented contact attempts. A documented status of “unable to reach” records the outcome of outreach rather than the confirmed resolution of surveillance, and “active surveillance” denotes ongoing follow-up rather than a terminal status. Baseline repair history at enrollment (prior EVAR in 1337 patients and prior open repair in 407 patients) is shown in Table 2 and is reported descriptively, not as an administrative status.

**Table 3.** Documented administrative status through April 2026 for all 8464 enrolled patients (STAIR AAA^a^ Registry, December 2022 through December 2024)^b^.

Documented administrative status (mutually exclusive)	Patients, n (%)
Active surveillance at April 2026	4188 (49.5)
Managed outside health system (transfer of care)	2709 (32.0)
No further protocolized STAIR surveillance required (advanced age or comorbidity)	623 (7.4)
Deceased	387 (4.6)
Refused further follow-up	293 (3.5)
Unable to reach despite repeated outreach	264 (3.1)
Total	8464 (100)

a STAIR AAA: System to Track Abnormalities of Importance Reliably Abdominal Aortic Aneurysm.

b Documented administrative status is provided for all 8464 patients in the STAIR AAA Registry, with a documented administrative status documented through April 2026 (enrollment period from Dec 2022 to Dec 2024). Each patient is assigned a single, mutually exclusive administrative status, and the categories sum to the full enrolled cohort of 8464 (100%); percentages are rounded and may not sum to exactly 100%. Baseline repair history at enrollment—prior endovascular aneurysm repair (1337) and open-repair (407)—is shown separately in Table 2 as a descriptive characteristic and is not an administrative status. A documented status of “unable to reach” (3.1%) reflects the outcome of repeated outreach rather than confirmed resolution of surveillance, and “active surveillance” (49.5%) denotes enrolled patients who had not reached any terminal administrative status at the April 2026 cutoff and were continuing under scheduled surveillance; this category is defined as the complement of the terminal status categories.

### Engagement Intensity After Enrollment

Among the 7018 patients (82.9% of the enrolled cohort) with observable EHR activity in both the before and after enrollment periods, the median duration of observable EHR history was substantially longer before STAIR enrollment than after: 15.35 (IQR 8.44‐22.40) years before enrollment versus 1.68 (IQR 0.79‐2.41) years after, reflecting that postenrollment follow-up was bounded by the duration of the STAIR program. Although the absolute number of EHR events was lower after enrollment than before, median 9 (IQR 4‐14) events versus 35 (IQR 16‐65) events, the time-normalized event rate more than doubled after enrollment, increasing from 2.82 (IQR 1.61‐4.41) events per year before enrollment to 6.64 (IQR, 4.59‐9.66) events per year after enrollment. This pattern is consistent with intensified surveillance contact around and following entry into the centralized program; however, this comparison is susceptible to indexing (ascertainment) bias, because enrollment is frequently precipitated by an acute or incidental imaging event that itself generates a cluster of immediate workup, and the postenrollment window is far shorter and more clinically concentrated than the multiyear pre-enrollment baseline (see “Limitations” section).

### Elective Repair Activity

During the study period (extending from the fourth quarter of 2022 through the fourth quarter of 2024), a total of 351 elective AAA repairs were performed within the health system, including 260 cohort patients and 91 noncohort patients (Table 4).

**Table 4.** Within-system AAA^a^ repair activity by cohort status, Geisinger Health System, Quarter 4 of 2022 through Quarter 4 of 2024^b^.

Repair type	Cohort patients, n	Noncohort patients, n	Total
Elective AAA repair	260	91	351
Ruptured AAA repair	0	28	28

a AAA: abdominal aortic aneurysm.

b AAA repair activity within the health system during the study period (Quarter 4 of 2022 through Quarter 4 of 2024). “Cohort” denotes patients enrolled in the STAIR (System to Track Abnormalities of Importance Reliably) Surveillance Registry at the time of repair; “noncohort” denotes patients undergoing repair within the health system who were not previously enrolled at the time of presentation, typically those presenting acutely or transferred from external facilities. Because noncohort status is defined partly by acute or transfer presentation, the cohort and noncohort groups are not comparable; these counts are descriptive only and must not be read as evidence of rupture prevention or clinical efficacy.

### Surveillance Imaging Usage

Among the 6951 patients with a recorded surveillance imaging study, duplex ultrasonography was the modality for 6122 (88.1%) and CT-based imaging for 829 (11.9%), comprising CT of the abdomen or pelvis in 180 (2.6%) and CT angiography in 649 (9.3%) patients; no MR angiography was used (0%) (Table 5).

**Table 5.** Surveillance imaging modality usage, STAIR AAA^a^ Registry, December 2022 through December 2024^b^.

Imaging modality	Patients, n (%)
Duplex ultrasonography	6122 (88.1)
Computed tomography: abdomen or pelvis	180 (2.6)
Computed tomography angiography	649 (9.3)
Magnetic resonance angiography (MRA)	0 (0)

a STAIR AAA: System to Track Abnormalities of Importance Reliably Abdominal Aortic Aneurysm.

b Surveillance imaging modalities used for ongoing AAA monitoring among patients enrolled in the STAIR AAA Registry between Dec 2022 and Dec 2024. Counts and percentages reflect the surveillance imaging modality recorded per patient among the 6951 enrolled patients with a documented surveillance imaging study; patients without a recorded surveillance imaging study (eg, those referred directly to vascular surgery, undergoing repair, or reaching an early administrative status) are not included. No MRA was used.

### Urgent Repair Volume

No ruptured AAA repairs occurred among cohort patients during the study period (Table 4). A total of 28 ruptured AAA repairs were performed among noncohort patients during the study period. These counts must not be interpreted as evidence that surveillance prevented ruptures. Because noncohort patients are defined as those presenting acutely or who were transferred from external facilities, the concentration of ruptures in the noncohort group is an expected structural consequence of the cohort definition and is subject to severe selection bias and immortal-time bias. No causal or comparative inference about rupture prevention can be drawn from these descriptive counts.

## Discussion

### Principal Findings

In a large integrated health system, a centralized digital surveillance program with NLP-based screening of radiology reports identified 8464 patients with AAAs through 4 complementary pathways, assigned guideline-based surveillance through standardized centralized clinical review, and documented an administrative status through April 2026 for all 8464 enrolled patients (a documented status includes categories such as unable to reach and active surveillance, which record outreach outcomes or ongoing follow-up rather than a confirmed resolution). Automated identification predominated, with 88.0% (7448/8464) of patients entering the registry through problem-list queries or NLP of radiology reports. Duplex ultrasonography was the predominant surveillance modality, consistent with image-stewardship considerations. The program was operationalized using existing clinical personnel infrastructure and was supported by a single full-time coordinator and an internally developed NLP case-identification model. Institutional informatics, software-engineering, and information-technology resources developed and maintained the NLP pipeline, supported EHR data integration, and operated the registry-tracking infrastructure. Compared with the prior decentralized model in which AAA surveillance relied on individual clinicians and inconsistent manual follow-up across departments, centralization required a single full-time coordinator and did not require new clinical positions beyond this role; previously fragmented surveillance work distributed across multiple services was consolidated into the centralized program rather than being added to existing workloads.

### Comparison With Prior Work

The predominance of problem-list and NLP pathways in our cohort (88.0%, 7448/8464) reinforces prior reports that most AAAs are detected incidentally outside dedicated vascular care and that reliance on clinician-initiated recognition and follow-up is vulnerable to inconsistency and loss to follow-up [1,2,7-9]. Prior studies have demonstrated the feasibility of AI-based aneurysm detection and measurement using radiological texts and imaging data [11,12]. This work extends 3 streams of recent literature. First, DiLosa et al [14] used NLP across 7 years of imaging reports at a single academic institution to identify 1424 patients with AAA and reported that 61% were not enrolled in active surveillance, and Boitano et al [13] described a nurse-navigator-run program that paired a commercial NLP system with EHR review to identify 6495 patients with AAA across an 11-year look-back at a tertiary academic hospital; the STAIR program extends these models by adding centralized clinical-review and registry-tracking layers over the NLP pipeline at a system level, enabling all 8464 identified patients to undergo centralized review and receive an initial surveillance or management assignment. Second, Kartsonis et al [16] demonstrated that transformer-based NLP models can flag and measure aortic diameters in radiology reports with high accuracy, and Flanagan et al [17] recently showed that locally hosted large language models can extract aortic information from abdominal imaging reports with an accuracy approaching 0.96 without task-specific training; the internally developed NLP detection model used in our program integrates with 3 additional identification pathways (problem-list query, clinician referral, lost-to-follow-up sweeps) to broaden case capture beyond what any single detector achieves and to provide a system-level surveillance infrastructure independent of the underlying detection algorithm. Third, Wilson et al [15] reported imaging-surveillance adherence of 67.7% among patients flagged by an AI-based algorithm; the STAIR program reports a documented administrative status for all 8464 enrolled patients, although the 2709 (32.0%) patients who transitioned to outside-system care had downstream surveillance adherence that could not be verified within our institution. Notably, 59.0% of identifications in our cohort came from problem-list queries, a structured-data pathway, not AI, underscoring that AI-assisted surveillance in current practice remains complementary to standard structured EHR querying rather than a replacement for it.

### Limitations

Several limitations merit consideration. First, the descriptive design and absence of a preimplementation comparator preclude causal inference regarding changes in clinical outcomes or health care usage. Observed elective and urgent repair volumes are reported descriptively and may reflect referral patterns, regionalization of care, and secular trends in AAA management. Second, the cohort is demographically homogeneous (White: 8311/8464, 98.2% and non-Hispanic: 8370/8464, 98.9%), reflecting the underlying patient population of a large rural integrated health system in central and northeastern Pennsylvania. NLP-based detection models can be sensitive to local documentation patterns and underlying population characteristics, and external validation in more diverse populations and care settings is required before generalizing operational performance. Third, long-term clinical outcomes were unavailable for the 2709 (32.0%, N=8464) patients who transitioned to management outside the health system, introducing potential follow-up bias. The program documented these transitions to distinguish them administratively from patients lost to follow-up despite repeated outreach, but downstream surveillance adherence outside the health system could not be confirmed. This high proportion of outside-system transfers is a primary source of potential outcome misclassification, and any interpretation of long-term surveillance findings must be conditioned on this incomplete ascertainment. Fourth, the case-identification model was validated within a single integrated health system on a small held-out gold-standard set of 99 imaging studies (95% CIs: precision 0.92‐1.00; recall 0.81‐0.95; and specificity 0.82‐0.99). This set underrepresented negative cases, so its AAA prevalence (71/99) exceeded that of an unselected screening population; because sensitivity and specificity are independent of prevalence whereas positive predictive value is not, the precision estimate (0.98) is the metric most likely to be optimistic in lower-prevalence settings. In addition, the gold-standard studies were not consecutively sampled but were drawn from reports surfaced or adjudicated within the STAIR workflow, which enriched the set for borderline cases and underrepresented easily classified studies with no AAA-related language; all reported metrics may therefore not transfer directly to the full consecutive imaging population, and the small sample further limits the precision of the estimates. Detection performance is also sensitive to local radiology reporting conventions and documentation templates. Prospective evaluation across multiple institutions and reporting environments, using a comprehensive, consecutively sampled imaging cohort, is needed to confirm real-world performance and generalizability. Fifth, cohort integrity was independently verified by an automated CT-based measurement system only for the 2894 patients with available CT imaging. For ultrasound-only patients, registry inclusion relied on centralized clinical review of original reports without an independent imaging-based confirmation. Sixth, the program depended on internally developed NLP and registry-tracking infrastructure; replication at another institution would require comparable software-engineering and informatics capacity to build and maintain an equivalent pipeline, in addition to the single full-time clinical coordinator described here. Seventh, aortic-diameter measurements were derived from heterogeneous imaging modalities and reports, introducing variability in aneurysm sizing. Eighth, the program enrolled all patients meeting surveillance thresholds regardless of age and comorbidity profile (except the age-90 transition). Active surveillance of incidentally detected small AAAs in older patients with limited life expectancy may yield limited long-term clinical benefit while consuming surveillance capacity, and future iterations could incorporate age- and comorbidity-stratified enrollment criteria to focus surveillance on patients most likely to benefit. Ninth, the comparison of pre-enrollment versus postenrollment EHR event rates is subject to ascertainment (indexing) bias: enrollment is frequently precipitated by an acute or incidental imaging event that generates a cluster of immediate workup, and the postenrollment window (median 1.68, IQR 0.79-2.41 y) is far shorter and more clinically concentrated than the 15.35-year pre-enrollment baseline, so the observed increase in event rate is descriptive of intensified contact around enrollment rather than evidence of a program-induced increase in engagement. Lastly, the within-system repair comparison by cohort status (Table 4), including the absence of ruptures among cohort patients, is structurally confounded by the cohort definition and by selection and immortal-time bias; it is presented descriptively and supports no causal or comparative inference about rupture prevention or clinical efficacy.

### Future Directions

Future work should evaluate the impact of centralized, AI-assisted surveillance on long-term clinical outcomes, including aneurysm rupture, mortality, and timeliness of elective repair, ideally in designs that incorporate preimplementation comparators or contemporaneous control populations. External validation of the internally developed NLP model using radiology report datasets drawn from multiple institutions, together with prospective evaluation of program performance in more demographically diverse patient populations, will be important to establish generalizability. Specifically, we propose (1) prospective multicenter external validation of the NLP model on consecutively sampled imaging across institutions with differing reporting conventions and (2) comparative effectiveness studies contrasting the centralized program against standard decentralized surveillance pathways, using preimplementation comparators or contemporaneous controls and prespecified end points of surveillance adherence, timeliness of elective repair, rupture, and mortality. Ongoing assessment of the technical-overhead profile required for replication will also help inform whether similar surveillance infrastructure is feasible in lower-resource settings. The high proportion of patients transitioning to out-of-system care (2709/8464, 32.0%) also raises an equity consideration not directly addressed by the present descriptive design. Surveillance quality for these patients depends on the practices of external providers, which may vary, and centralized programs of this kind risk creating differential access to longitudinal AAA monitoring if downstream follow-up cannot be confirmed. Future iterations should consider mechanisms, such as bidirectional data exchange with referring providers, structured outreach to externally managed patients, or integration with regional health information networks, to extend surveillance benefits beyond the institution.

### Conclusions

This retrospective cohort study describes the design, implementation, and operational performance of a centralized, AI-assisted AAA surveillance program within a large, integrated health system. Within the limits of a descriptive design, the program supported population-level case identification through 4 complementary pathways, standardized guideline-based surveillance and management assignment through centralized clinical review, and a documented administrative status through April 2026 for all 8464 enrolled patients. These findings establish operational feasibility only; clinical effectiveness (including any effect on rupture, mortality, or surveillance adherence) is not demonstrated by this descriptive design and will require future comparative work.

## Abbreviations

AAA: abdominal aortic aneurysm
CT: computed tomography
CTA: computed tomography angiography
EHR: electronic health record
ESVS: European Society for Vascular Surgery
EVAR: endovascular aneurysm repair
NLP: natural language processing
STAIR: System to Track Abnormalities of Importance Reliably
SVS: Society for Vascular Surgery
